# Acute HIV-1 and SARS-CoV-2 Infections Share Slan+ Monocyte Depletion—Evidence from an Hyperacute HIV-1 Case Report

**DOI:** 10.3390/v13091805

**Published:** 2021-09-10

**Authors:** Guilherme B. Farias, Robert Badura, Carolina M. Conceição, André M. C. Gomes, Ana Godinho-Santos, Joel Laia, Pedro Rosmaninho, Diana F. Santos, Catarina Mota, Afonso R. M. Almeida, Susana M. Fernandes, Amelia C. Trombetta, Ana E. Sousa

**Affiliations:** 1 Instituto de Medicina Molecular João Lobo Antunes, Faculdade de Medicina, Universidade de Lisboa, 1649-028 Lisboa, Portugal; guilherme.farias@medicina.ulisboa.pt (G.B.F.); rbadura@medicina.ulisboa.pt (R.B.); carolina.conceicao@medicina.ulisboa.pt (C.M.C.); andre.gomes@medicina.ulisboa.pt (A.M.C.G.); agsantos@medicina.ulisboa.pt (A.G.-S.); joel.laia@medicina.ulisboa.pt (J.L.); pedro.rosmaninho@medicina.ulisboa.pt (P.R.); diana.santos@medicina.ulisboa.pt (D.F.S.); mcsilva@medicina.ulisboa.pt (C.M.); afonso.almeida@medicina.ulisboa.pt (A.R.M.A.); susanamfernandes@medicina.ulisboa.pt (S.M.F.); 2 Centro Hospitalar Universitário Lisboa Norte, Hospital de Santa Maria, 1649-028 Lisboa, Portugal

**Keywords:** acute HIV-1 infection, COVID-19, monocytes, slan, M-DC8, dendritic cells

## Abstract

Monocytes are key modulators in acute viral infections, determining both inflammation and development of specific B- and T-cell responses. Recently, these cells were shown to be associated to different SARS-CoV-2 infection outcome. However, their role in acute HIV-1 infection remains unclear. We had the opportunity to evaluate the mononuclear cell compartment in an early hyper-acute HIV-1 patient in comparison with an untreated chronic HIV-1 and a cohort of SARS-CoV-2 infected patients, by high dimensional flow cytometry using an unsupervised approach. A distinct polarization of the monocyte phenotype was observed in the two viral infections, with maintenance of pro-inflammatory M1-like profile in HIV-1, in contrast to the M2-like immunosuppressive shift in SARS-CoV-2. Noticeably, both acute infections had reduced CD14^low/−^CD16^+^ non-classical monocytes, with depletion of the population expressing Slan (6-sulfo LacNac), which is thought to contribute to immune surveillance through pro-inflammatory properties. This depletion indicates a potential role of these cells in acute viral infection, which has not previously been explored. The inflammatory state accompanied by the depletion of Slan+ monocytes may provide new insights on the critical events that determine the rate of viral set-point in acute HIV-1 infection and subsequent impact on transmission and reservoir establishment.

## 1. Introduction

The introduction of combined antiretroviral therapy (cART), in 1996, dramatically changed the prognosis of HIV-1 infection, but soon it was realized the need of complementary approaches to control viral persistency. Although the early start of cART in the acute HIV-1 infection reduces the viral set-point and immune-activation, it does not prevent the establishment of cell reservoirs of integrated provirus [1]. Therefore, it is of utmost importance to uncover the initial immune responses with impact on the viral dynamics.

The inflammatory environment is determinant for the dissemination and the establishment of HIV-1 infection, and monocytes are central in these processes [2]. In fact, monocyte responses have been emerging as main contributors for the pathogenesis of another acute infection, severe acute respiratory syndrome coronavirus 2 (SARS-CoV-2) [3]. This pathogen causes Coronavirus Disease 2019 (COVID-19), that can lead to respiratory failure and death in higher risk groups [4].

Monocytes and macrophages play a critical role in the development and homeostasis of all tissues and, together with dendritic cells, are critical for the initiation of an adaptive immune response [2]. The proper activation of the adaptive immune response is associated with better disease control in both the acute and chronic stages of HIV-1 infection [5,6,7,8]. In circulation, monocytes can be divided into three subsets: classical (CD14^high^CD16^−^), intermediate (CD14^high^CD16^+^), and non-classical (CD14^low/neg^CD16^+^). These subsets show transcriptomic differences that translate into specialization into different functions [9]. Due to their capability to produce inflammatory cytokines, and high mobility, non-classical monocytes are characterized as a pro-inflammatory population [10,11]. This subset can then be further divided into Slan positive (Slan^+^) and Slan negative populations [12,13,14]. Monocytes differentiate into macrophages and dendritic cells in the tissues [2,15]. Recently, the macrophage/monocyte phenotype has been better dissected, leading to the establishment of a pro-inflammatory phenotype (M1-like), expressing higher levels of CD80, CD86, and HLA-DR, or an immune-suppressive/wound healing phenotype (M2-like), expressing higher levels of CD204 and CD206, as extremes of a differentiation spectrum [16,17].

Monocyte/macrophage activation syndrome was reported in severe COVID-19 cases, with interferon-γ (IFNγ), interleukin-1 (IL-1), IL-6, tumor necrosis factor-α (TNFα), and IL-18 having central immunopathogenic roles in the hyper-inflammation [18]. Our lab demonstrated that the expansion of M2-like monocytes was significantly higher in patients with detectable SARS-CoV-2 plasma viral load and that systemic immune-regulatory myeloid cell phenotype was the main feature associated with the recovery of hospitalized patients [19].

Conversely, the pro-inflammatory state is a hallmark of chronic HIV-1 infection [20]. This is reflected in the upregulation of activation markers in classical monocytes and the expansion of non-classical monocytes [21]. This state persists even in treated individuals and is thought to contribute to the development of non-AIDS related co-morbidities [22].

The establishment of the inflammatory state occurs early in HIV-1 infection, as shown by the increase in activation markers like HLA-DR highlighting their contribution to CD4+ T-cell activation [6,23,24,25,26]. The data on the monocyte phenotype during the acute HIV-1 infection are relatively scarce due to the rarity of these patients. In fact, more than 50% of acute HIV infection [27] are asymptomatic, imposing serious limits on the clinical investigation of these initial stages. Therefore, the opportunity to investigate an acute HIV-1 infected patient, in parallel with a cohort of SARS-CoV-2 infected individuals allowed us to explore the monocyte phenotype upon exposure to two distinct viruses. We expect that this case report will inform the design of future studies aiming to better understand the contribution of the inflammatory response to the infection outcome.

## 2. Materials and Methods

### 2.1. Patients and Controls

The study involved an early acute HIV-1 infected patient at Fiebig stage II [28], a chronic HIV-1 infected individual at the time of diagnosis without exposure to antiretroviral therapy, and twenty patients with acute SARS-CoV-2 infection (confirmed by RT-PCR of nasopharyngeal swabs), all followed at the Centro Hospitalar Universitário Lisboa Norte (CHULN, Lisboa, Portugal). Eleven healthy individuals were included as controls. The clinical and epidemiological details are depicted in Table 1. The COVID-19 patients were previously included in a recently published study [19]. Clinical data were collected by the clinicians integrating the research team. Whole blood was processed immediately after sampling. There were no differences in sample handling or material used among patients and healthy controls. Informed consent was obtained from all participants and the study was approved by the Ethics committee of the CHULN/Faculdade de Medicina da Universidade de Lisboa/Centro Académico de Medicina de Lisboa.

### 2.2. Flow Cytometry

Multi-parameter flow cytometry was performed on whole blood immediately after sample collection. After erythrocyte bulk lysis, 10 million leukocytes were incubated at room temperature for 30 min with a panel of fluorochrome labelled antibodies: anti-Slan Vioblue, anti-CD141 BV510, anti-CD45 BV605, anti-HLA-DR BV650, anti-CD86 BV711, anti-PD-L1 BV785, anti-CD3 FITC, anti-CD19 FITC, anti-CD66b FITC, anti-CD14 PerCP-Cy5.5, anti-CD80 PE, anti-CD163 PE-dazzle594, anti-CD206 PE-Cy5, anti-CD123 PE-Cy7, anti-CD204 APC, anti-CD16 Alexa Fluor 700, and anti-CD1c APC-Cy7. The list of antibody clones is provided in Table S1. After fixation, cells were re-suspended in PBS and acquired in a Fortessa X-20 flow cytometer. The resulting flow cytometry data were compensated and analyzed with FlowJo software (Version 10.7; Tree Star, Inc., Ashland, OR, USA). The gating strategy used is represented in Figure S1. The monocytes, macrophages, and dendritic cells were defined by selecting cells expressing CD45 and not the lineage markers CD3, CD19, and CD66b (CD45^+^Lin^−^). In parallel, a tube to identify the main lymphocyte populations was performed including the following fluorochrome labelled antibodies: anti-CD27 BV421, anti-IgM BV510, anti-CD4 V500, anti-IgD BV605, anti-CD8 BV605, anti-HLA-DR BV650, anti-CD21 BV711, anti-CD45RO BV785, anti-CCR7 FITC, anti-CD3 PerCP-Cy5.5, anti-CD56 PE, anti-CXCR5 PE-dazzle594, anti-CD38 PE-Cy5, anti-CD19 PE-Cy7, anti-γδ PE-Cy7, anti-CD25 APC, anti-CD16 Alexa Fluor 700, anti-CD127 APC-efluor 780 (Table S1).

### 2.3. Unsupervised Analysis

For the unsupervised analysis we followed the workflow described by Melsen J. et al. [29]. After compensation, we selected the CD45^+^Lin^−^ cells and performed the analysis on the same number of events (72591) from each patient and healthy control. After transformation and normalization of the data, we performed dimensionality reduction with Uniform Manifold Approximation and Projection (UMAP) using the *uwot* package [30]. For clustering, the X-shift algorithm was applied to define the appropriate number of clusters for the dataset [31]. Afterwards, the data were clustered using FlowSOM [32]. The UMAP highlighting the 14 clusters obtained, as well as the expression of the different markers are shown in the Figure S2. All data analysis was performed in R (4.1.0), using Bioconductor (3.13) [33]. For data visualization the *pheatmap* and *tidyverse* packages were used. UMAPs were also generated on T cells, gating on CD3+ lymphocytes, and on B cells, gating on CD19+ lymphocytes, using 57,907 and 10,012 events from each patient and healthy control, respectively. The main T- and B-cell subsets were then defined manually (Figures S3 and S4, respectively), using FlowJo.

### 2.4. HIV-1 Read-Outs

Viral load was measured by real-time PCR performed on whole blood in EDTA, Cobas^®^ 6800/8000 (Roche, Basel, Switzerland), targeting the LTR and gag regions of HIV-1 genome, enabling quantification and subtypification among: HIV-1M (A-D, F-H, CRF01_AE, CRF02_AG), HIV-1O, HIV-1N. Detection of antibodies for HIV-1 and HIV-2, was made using a fourth generation ELISA (Cobas 8000; Roche, Basel, Switzerland). Antigenemia for p24 was measured using Evolis system (BioRad; Hercules, CA, USA). Finally, western blots were performed on Env, Pol and Gag for HIV-1 and 2, NewLavBlot 1 and NewLavBlot 2 assays in a Autoblot 3000 (BioRad).

### 2.5. SARS-CoV2 Plasma Viral Load

A droplet digital PCR (ddPCR) test kit (SARS-CoV-2 ddPCR Test Kit, Bio-Rad) was used to quantify SARS-CoV2 plasma viral load, on QX200™ ddPCR System (Bio-Rad), following manufacturer’s instructions. Total RNA was extracted from 560 µL of plasma (QIAamp^®^ Viral-RNA MiniKit, QIAGEN), and duplicates of 20 μL ddPCR reaction using 5 μL RNA were analyzed on QuantaSoft Analysis Pro (1.0.596). Plasma samples with N1 or N2 regions, or both regions detected were considered positive. SARS-CoV-2 RNA concentrations (cp/mL) were calculated considering the extracted volume of plasma.

## 3. Results

Our aim was to compare the monocyte and dendritic cell phenotype in acute HIV-1 and SARS-CoV-2 infections.

We were able to investigate an early hyper-acute HIV-1 infected individual, classified as Fiebig stage two, possessing high levels of plasma viral load and detectable p24 antigenemia in the absence of specific HIV antibodies [28]. The patient featured fever and asthenia preceding 13 days the diagnosis, and elevated aminotransferases, compatible with a HIV-induced hepatitis and high inflammatory response. The comparison was performed with a cohort of 20 acute SARS-CoV-2 patients, included in a previous study [19]. As controls, we enrolled 11 healthy subjects and a treatment naïve chronic HIV-1 patient, asymptomatic despite an advanced disease stage as illustrated by the low CD4+ T-cell count (Table 1).

Both the acute HIV-1- and SARS-CoV-2-infected patients showed signs of inflammation, namely elevated C reactive protein (CRP) and ferritin levels, as expected in an acute viral infection, though IL-6 was only elevated in SARS-CoV-2-infected patients. COVID-19 was associated with neutrophilia with lymphopenia, as reported before [3], which was not observed in the acute HIV-1-infected patient (Table 1). Despite a normal lymphocyte count, we observed a reduction of circulating CD4+ T cells in the acute HIV-1 patient, yet in the absence of CD8+ T-cell expansion (Table 1). These features were observed in the chronically HIV-1-infected patient, who showed the highest inversion of the CD4/CD8 ratio (Table 1). Monocyte counts were higher in HIV-1 as compared to SARS-CoV-2-infected individuals and the controls (Table 1).

**Figure 1:**
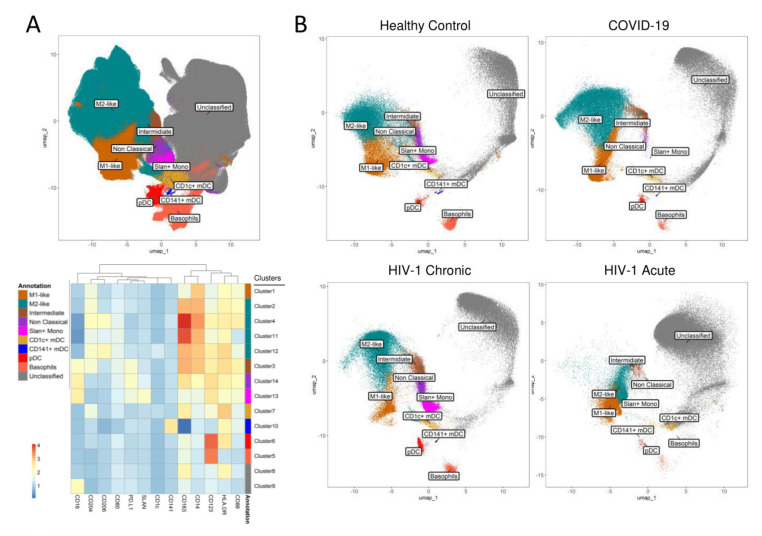
Profiling of the mononuclear cells compartment in HIV-1 and SARS-CoV-2 infections using unsupervised analysis. (**A**) UMAP of the CD45^+^Lin^−^ dataset of all samples with the heatmap showing relative marker expression of the clusters obtained with the FlowSOM clustering algorithm and the manual annotation of the main subsets. (**B**) Relative subset distribution in representative individuals from the healthy control and COVID-19 groups, as well as in the untreated HIV-1 acute and HIV-1 chronic patients.

In order to explore the impact of acute HIV-1 infection on the circulating monocytes and dendritic cells, we performed an unsupervised analysis of a panel of M1 and M2 markers by flow cytometry, using UMAP and clustering with the FlowSOM algorithm, obtaining 14 clusters (Figure 1, Figures S1 and S2). To facilitate the interpretation the 14 clusters obtained were further annotated into 10 subsets based on marker expression shown in Figure S2, defining the major monocyte and dendritic cell populations (Figure 1A).

**Figure 2:**
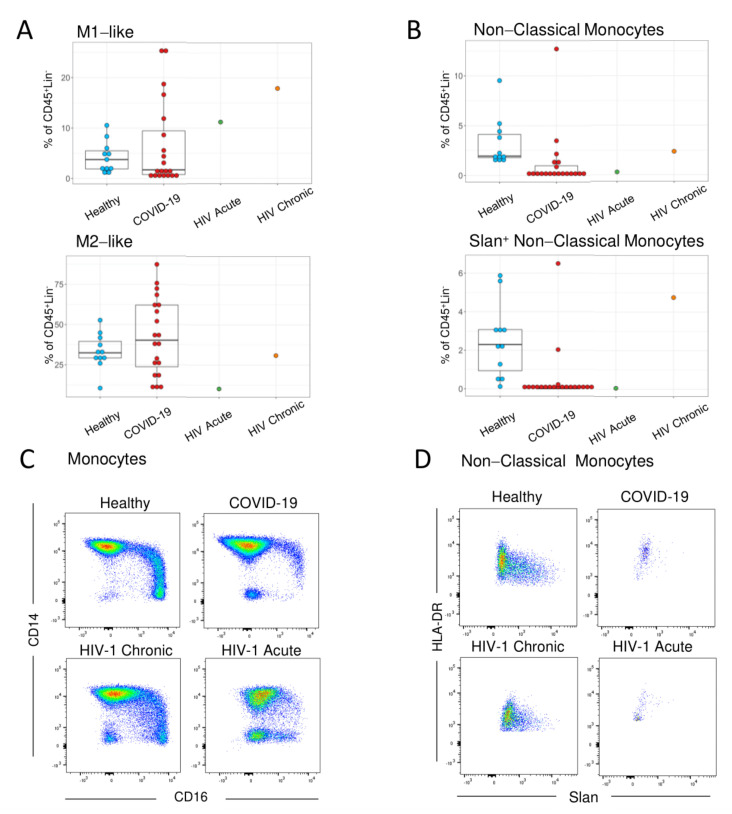
Shift to M1-like profile and loss of Slan+ Monocytes in acute HIV-1 infection. (**A**,**B**) Boxplots showing the frequencies and the medians of the identified subsets using FlowSOM, as shown in Figure 1. Each dot represents one subject. (**C**,**D**) Illustrative dot plots of the manual analysis performed in representative individuals from the healthy control and COVID-19 groups, as well as in the HIV-1 acute and untreated HIV-1 chronic patients showing the expression of CD14 and CD16 in monocytes (**C**); and the expression of HLA-DR and Slan in the non-classical (CD14^low/−^CD16^+^) monocytes (**D**).

Classical monocytes represent a key population of interest to be studied in acute viral infections, given their wide spectrum of inflammatory and homeostatic functions [34]. The relative population distribution in the acute HIV-1 patient revealed a two-fold increase in the pro-inflammatory M1-like phenotype in classical monocytes, defined as CD14^high^ CD16^−^ CD86^+^ CD80^+^ CD163^low^, when compared to controls (Figure 1B and Figure 2A). COVID-19 patients showed the opposite trend, having an increase in the more immunomodulatory M2-like classical monocytes, defined as CD14^high^ CD16^−^ CD163^high^ CD204^+^ CD206^+^, with a reduction in the M1-like phenotype (Figure 1B and Figure 2B). The chronic HIV-1 patient followed the trend of the acute HIV-1 patient, with a more pronounced increase in M1-like classical monocytes (Figure 1B and Figure 2A). This shift in the balance between M1 and M2 phenotypes highlights the high plasticity of responses in these cells, as HIV-1 infection was associated with increased expression of immune activation and inflammation markers, while SARS-CoV-2 increased the markers associated with immunomodulation.

Interestingly, another population that seemed to be highly affected in acute HIV-1 infection was the non-classical (CD14^low/−^ CD16^+^) monocytes. Non-classical monocytes are known to be readily able to leave circulation to exert several pro-inflammatory functions in the tissues. This population was decreased in both acute HIV-1 and SARS-CoV-2 infections, with the Slan+ subset being the most affected (Figure 1B and Figure 2B). Using manual analysis, we confirmed that there was a noticeable loss of non-classical monocytes in both acute HIV-1 and SARS-CoV-2 infections (Figure 2C), with the Slan+ population being depleted in both cases (Figure 2D). It is important to note that the chronic HIV-1 patient did not show a decrease of non-classical monocytes, and presented an expansion of the Slan+ subset, which is in accordance with previous literature on chronic viremic HIV-1 patients [35,36].

**Figure 3:**
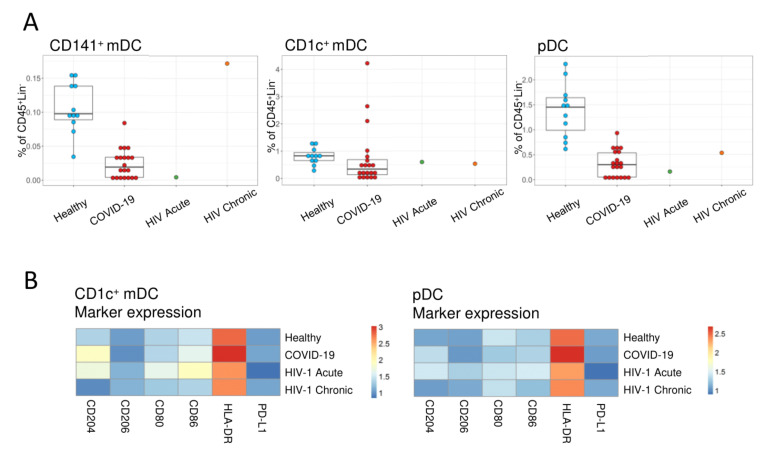
Circulating dendritic cells in acute HIV-1 infection. (**A**) Boxplots showing the frequencies and the medians of the identified population. Each dot represents one subject. (**B**) Heatmap showing the expression of co-stimulatory and activation markers within CD1c+ mDCs and pDCs for the HIV-1 acute and untreated HIV-1 chronic patients, as well as the healthy control and COVID-19 groups. CD141+ mDC marker expression is not shown given the low number of events.

Regarding myeloid dendritic cells (mDC), the myeloid subset expressing CD141 (CD141+ mDC), was reduced in both acute HIV-1 and SARS-CoV-2 infections, whereas the CD1c+ mDC subset was relatively preserved (Figure 1B and Figure 3A). However, there were differences in phenotype of CD1c+ mDCs, namely higher levels of activation/co-stimulatory markers CD80 and CD86 in the acute HIV-1 as compared to SARS-CoV-2-infected patients (Figure 3B). The plasmacytoid dendritic cells (pDC) were also reduced in acute HIV-1 and SARS-CoV-2 patients (Figure 3A), without any noticeable alterations in marker expression (Figure 3B). The reduction in circulating dendritic cells and the reduced expression of activation markers are in agreement with previous studies that revealed an impaired function of dendritic cells in COVID-19 [37].

Both monocytes and dendritic cells play an important role in the initiation of the adaptive immune responses. Therefore, we performed a parallel evaluation of the T- and B-cell compartments with a dimensionality reduction by UMAP on the CD3+ (Figure S3) and CD19+ lymphocytes (Figure S4), respectively.

We documented CD4+ and CD8+ T-cell activation in both acute infections, associated with expansion of central and effector memory subsets, and increased expression of CD38 and HLA-DR in the CD8+ T cells, particularly in the hyper-acute HIV-1 infected patient (Figure S3). As, expected the untreated HIV-1 infected patient featured a marked depletion of CD4+ T cells and expansion of CD8+ T cells, accompanied by contraction of the naïve compartment and terminally-effector T-cell differentiation (Figure S3). Importantly, we observed a clear expansion of circulating plasmablasts in both acute HIV-1 and SARS-CoV-2 infections, supporting the mounting of specific antibody responses, which were not detected in chronic HIV-1 infection and healthy controls as expected. Moreover, both acute infections featured expansion of memory switched and unswitched B-cell populations (Figure S4). The memory B-cell subsets were already lost in the advanced chronically infected HIV-1 infected patient, as previously described [38].

## 4. Discussion

Our data illustrate the plasticity of the monocyte and dendritic cell phenotype in acute viral infections, as seen by the different changes in the M1-M2-like balance between HIV-1 and SARS-CoV-2 infection in classical monocytes. At the same time, a non-previously described depletion of the Slan+ subset of non-classical monocytes was found in the two acute infections. Thus, this HIV-1 acute case report provides new lines to further investigate the myeloid compartment in future cohort studies.

M1-like classical monocyte expansion was observed in the acute HIV-1 patient, whereas in SARS-CoV-2 infection the immunomodulatory M2-like classical monocytes dominated in our study. The latter might be a negative feedback loop to an earlier excessive inflammatory response seen in SARS-CoV2 [19]. Importantly, this was not the case in HIV-1, where the M1-like expansion further increased in the case of untreated chronic HIV-1 infection. Moreover, this pro-inflammatory state persists even after starting cART and is thought to contribute to non-AIDS related co-morbidities [20,39]. It is likely that the persistent inflammation during the acute HIV-1 infection contributes to the dissemination and persistence of HIV-1 [7,40]. Monocytes have been shown to upregulate activation markers during the sharp increase in viremia observed very early upon HIV-1 infection, such as in Fiebig stage II [24]. Given the role of these cells as inflammatory cytokine producers [41] and stimulators of the adaptive immune responses, the M1-like population could contribute to the persistent immune activation that leads to the depletion of CD4+ T cells throughout the chronic infection [42].

The rise in M2-like phenotype in SARS-CoV-2 infection has been previously described, particularly in patients requiring hospital admission [43,44]. This has been mainly associated with severe disease [43,44], although the expansion of this immunomodulatory population might be a response to the extensive tissue damage and inflammation that occur in severe SARS-CoV-2 disease. As such, we recently reported that COVID-19 recovery is associated with the maintenance of this M2-like phenotype expansion [19].

In spite of these differences, our study revealed that a reduction of non-classical monocytes was shared by both acute HIV-1 and SARS-CoV-2 infections, with a striking depletion of Slan+ non-classical monocytes. Non-classical monocytes are able to quickly leave circulation to sites of inflammation, where they produce inflammatory cytokines such as TNF-α, IL-1β, and IL-12 [45] and are involved in viral response. The Slan+ subset of non-classical monocytes has been shown to produce high amounts of TNF-α and can show a DC like phenotype with a high capacity for co-stimulation of CD4+ T cells [13,46,47]. Although these cells have been studied in the context of chronic inflammation, as in psoriasis [48], their potential role in early responses such as in acute viral infection is still unclear. Using both unsupervised and supervised analysis, we show here, for the first time, that Slan+ monocytes are depleted in acute HIV-1 infection. The loss of this population could be due to the rapid recruitment of these cells to sites of infection, and the persistence of this depletion may be due to the slow reconstitution rate of these cells [49]. The Slan+ monocyte depletion was not apparently related to the plasma viral load, since it occurred in both viremic and non-viremic COVID-19 patients in our study. Of note, the depletion of this population does not seem to be related to age, since our acute HIV-1 patient is younger than our acute SARS-CoV-2 infected patients and the same Slan+ monocyte depletion was observed. Additionally, an expansion of this subset was observed in our viremic chronic HIV-1 infected patient. In SARS-CoV-2, these cells were suggested to play a role in the rampant inflammation that leads to the worsening of COVID-19, and have been shown to be reduced by us and in another study [19,44]. In acute HIV-1 infection, these cells might partake in the complex set of immune responses and activation, contributing to the viral set-point, through their ability to activate CD4+ T cells [13], the primary targets of HIV-1 infection. On the other hand, these cells have been confirmed to have a role in chronic HIV-1 infection, where they were found to be expanded and responsible for the overproduction of TNF-α and Il-β [35,36], which was confirmed in the current study.

In both acute infections we observed a loss of circulating CD141+ mDC and pDC, as previously reported [37,44,50], though the acute HIV-1 patient appeared to feature a more co-stimulatory profile, in contrast with the COVID-19 cohort. Nevertheless, we found evidence of mounting of B- and T-cell responses in both acute infections, as expected [8,51,52,53,54].

Monocytes, macrophages, and DC responses are considered the main determinants of the viral set-point upon HIV-1 infection, being associated to different disease outcome [24,55,56]. Early hyper-acute and untreated chronic HIV-1-infected individuals are increasingly rare due to the 40 decades of work to control HIV-1 transmission. Therefore, our study is limited by the reduced number of these samples and should be further validated in larger acute HIV-1 cohorts. Nevertheless, it does suggest a role for Slan+ monocytes in acute viral infection pathogenesis and highlight the capacity of monocytes to respond specifically to different viral infections.

In conclusion, our study revealed depletion of Slan+ monocytes in acute HIV-1 and SARS-CoV-2 infections, despite the opposite shifts in classical monocyte polarization. These results prompt future studies on the potential role that this population might have in the complex immune activation state governing the progression of HIV-1 infection and other viral infections.

## Figures and Tables

**Table 1:** Clinical and routine laboratory data from patients and healthy controls.

	HIV-1 Acute	HIV-1 Chronic	COVID-19 *	Healthy Controls
Number (males)	1 (0)	1 (1)	20 (17)	11 (9)
Age (years)	22	49	55.5 (39–65)	58 (39–65)
Time from symptoms start (days)	13	NA	8.5 (5–11)	NA
CRP (mg/dL)	2.60	0.37	8.8 (4.85–25.5)	ND
PCT (ng/mL)	0.48	ULoD	0.16 (0.11–0.38)	ND
Ferritin (ng/mL)	412	262	939 (402–1906)	ND
Interleukin 6 (pg/mL)	1.5	2.8	18 (4.5–36)	0.85 (0.24–1.6)
Lymphocytes/μL	1620	3130	920 (845–1662)	1940 (1423–2200)
CD4+ T cells/μL	364	210	247 (133–392)	768 (544–998)
CD8+ T cells/μL	503	863	145 (81.2–262)	414 (158–577)
CD4/CD8 ratio	0.72	0.24	1.78 (0.93–2.50)	1.95 (1.53–4.17)
Neutrophils/mL	1830	2290	4251 (2413–6917)	3228 (2521–6390)
Lymphocytes/neutrophils ratio	0.89	1.37	0.23 (0.15–0.5)	0.51 (0.47–0.61)
Monocytes/mL	810	720	349 (223–537)	398 (275–733)
Basophils/mL	10	50	20 (9.7–35)	32 (16–63)
Eosinophils/mL	30	200	13 (6.7–56)	115 (96–297)
SARS-CoV-2 Plasma Viral Load ^#^ (RNA cps/mL)	NA	NA	112 (24–498)	NA
HIV-1 Plasma Viral Load (RNA cps/mL)	1,320,000	57,200	NA	NA

Values expressed as medians (interquartile range) unless otherwise specified. CRP: C reactive protein; PCT: procalcitonin; NA: not applicable; ND: not done; ULoD: under limits of detection. * COVID-19-associated co-morbidities: arterial hypertension 9 patients (45%); diabetes type II 6 patients (30%); obesity 6 patients (30%); lung emphysema 2 patients (10%); no co-morbidities in HIV-1 infected patients; ^#^ Quantified in the 13 out of 20 COVID-19 patients with detectable SARS-CoV-2 plasma viral load.

## Data Availability

The data that support the findings of this study are available on request to the corresponding author.

## Supplementary Materials

The following are available online at https://www.mdpi.com/article/10.3390/v13091805/s1. Figure S1: Flow cytometry gating strategy. Figure S2: Unsupervised analysis of monocytes and dendritic cells. Figure S3: Unsupervised analysis of T cells. Figure S4: Unsupervised analysis of B cells. Table S1: Flow cytometry antibodies.
