# Supplementary material for: Acute HIV-1 and SARS-CoV-2 Infections Share Slan+ Monocyte Depletion—Evidence from an Hyperacute HIV-1 Case Report
Source: Viruses. 2021 Sep 10;13(9):1805. doi: 10.3390/v13091805 (PMC8473247; doi:10.3390/v13091805)
Supplement: Supplementary file 1 [file viruses-13-01805-s001.zip › viruses-1344223-Supplementary (5).pdf]

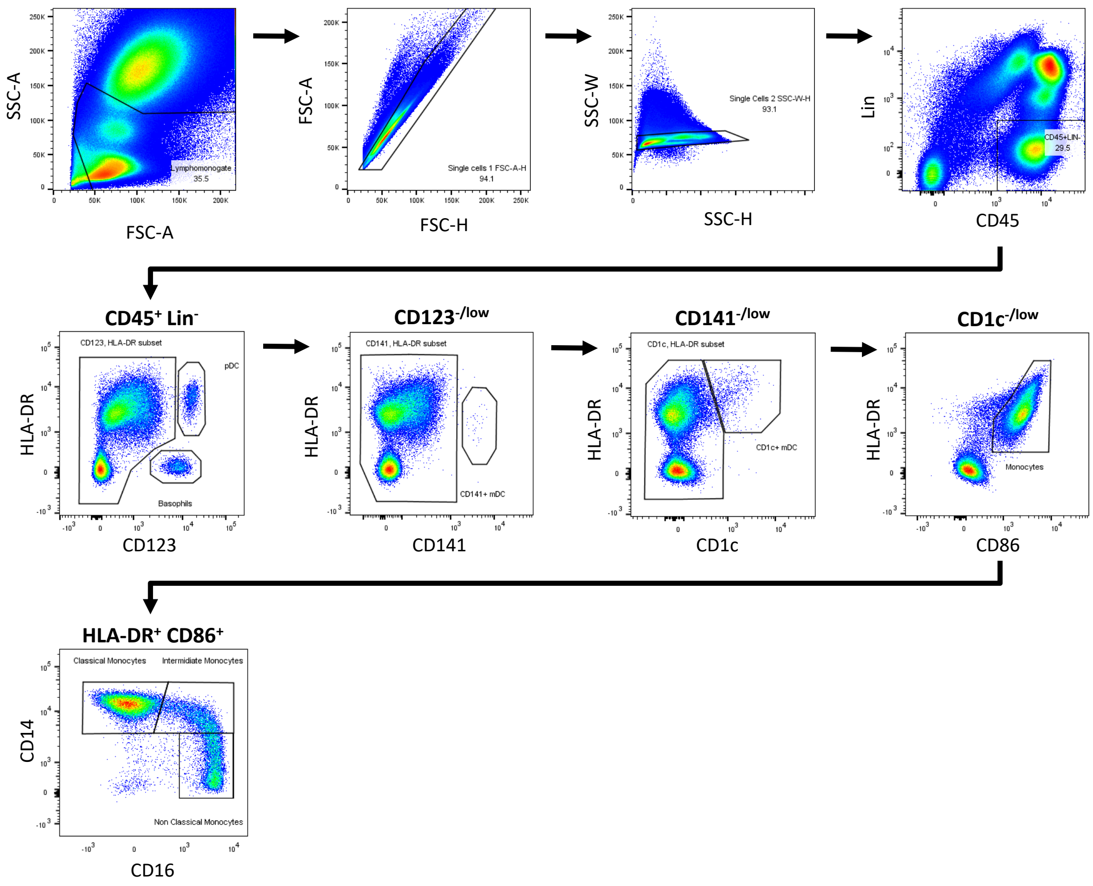

Figure S1. *Flow cytometry gating strategy.* Illustrative gating strategy used to identify monocyte and dendritic cells from whole blood, starting from 10 million leukocytes. Dot plots showing data from a representative healthy control.

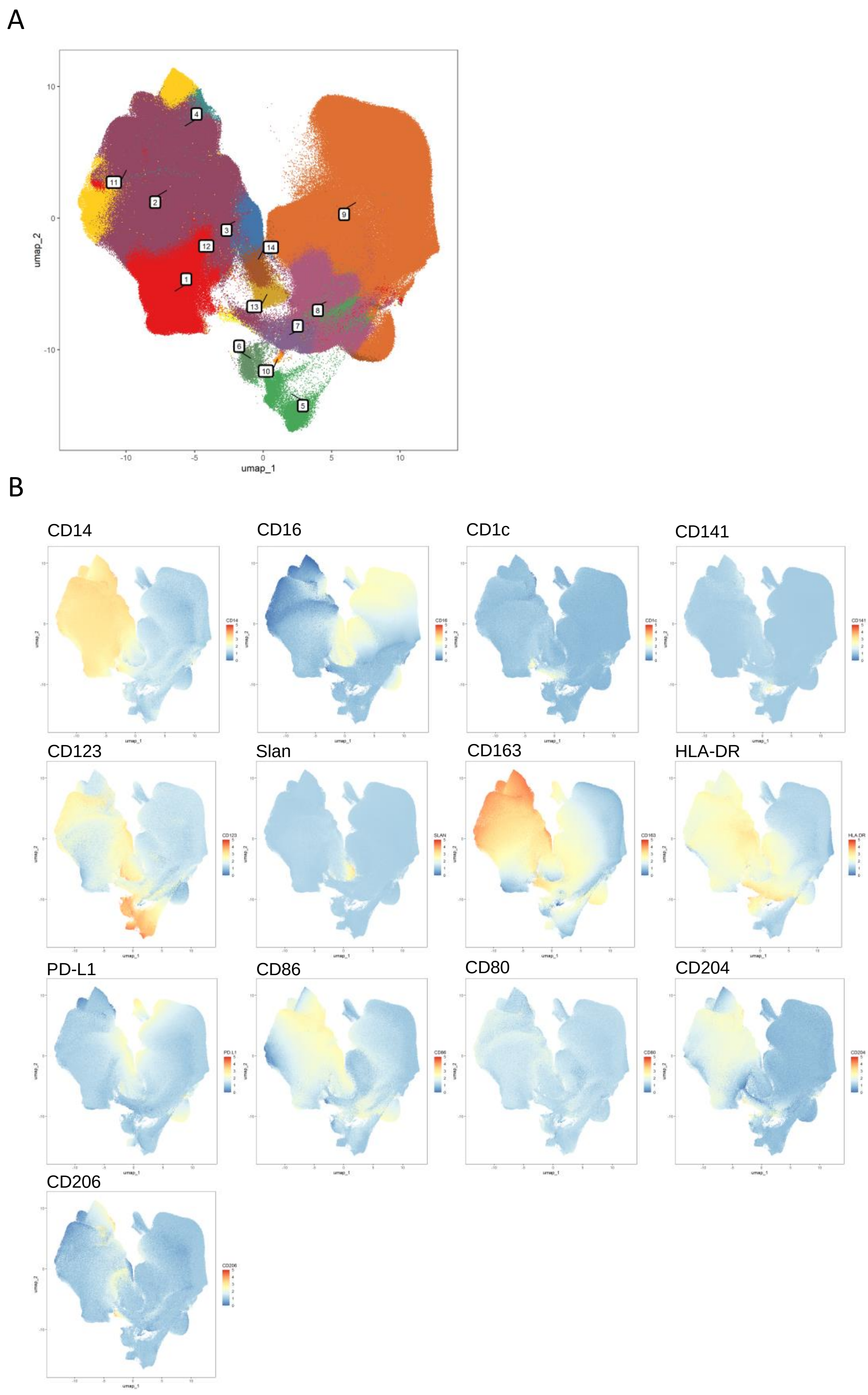

Figure S2. *Unsupervised analysis of monocytes and dendritic cells* (**A**) UMAP of the CD45+Lin- dataset of all samples showing the 14 clusters obtained with FlowSOM. (**B**) Marker expression within the UMAP.

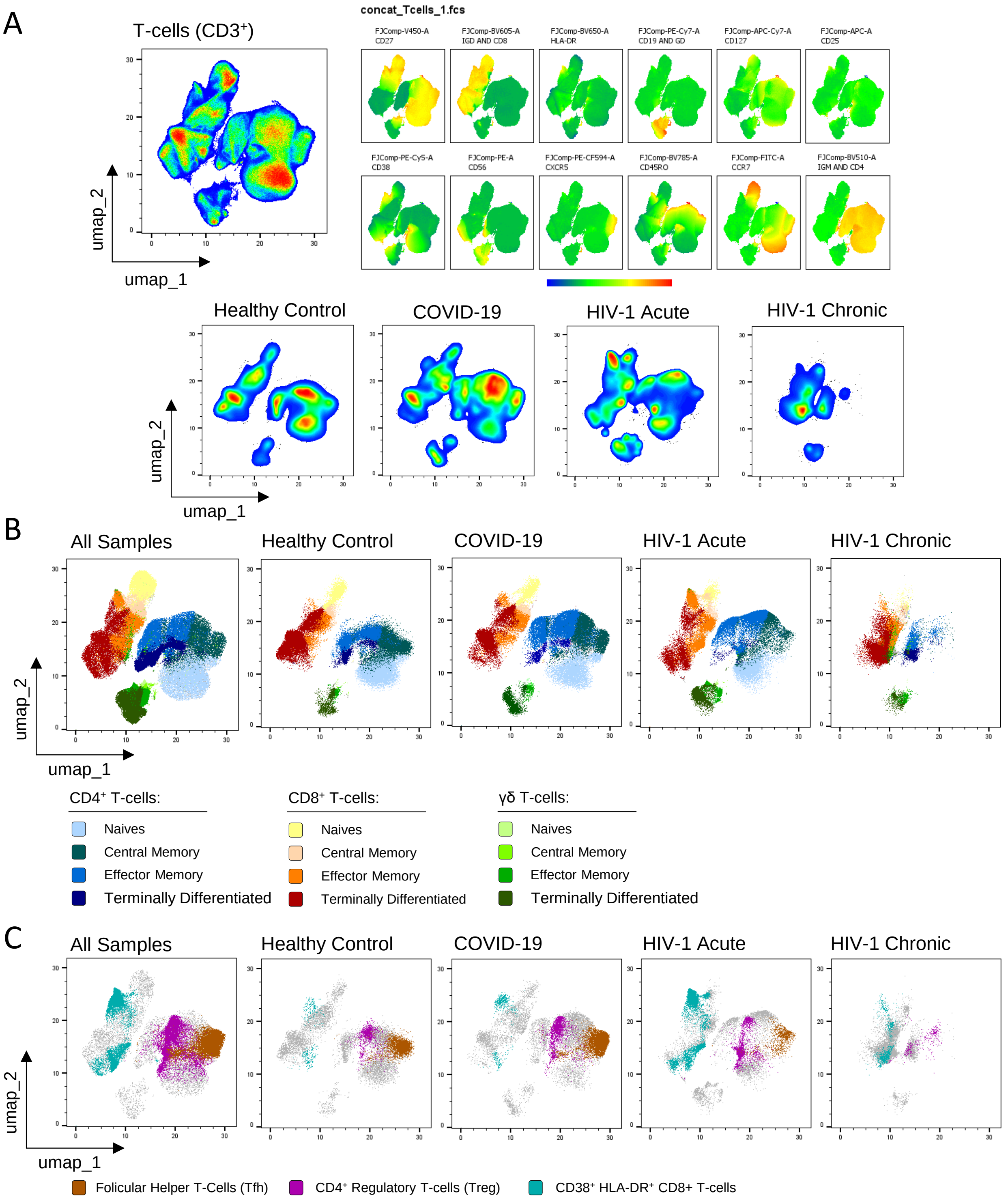

Figure S3. *Unsupervised analysis of T-cells*. **(A)** Marker expression within the UMAP of the CD3^{+} cells and relative event distribution in a representative individual from the healthy control and COVID-19 groups, as well as in the untreated HIV-1 acute and HIV-1 chronic patients. **(B-C)** Manually annotated T-cell subsets: **(B)** Naïve (CCR7^{+}CD45RO^{-}), Central Memory (CCR7^{+}CD45RO^{+}), Effector Memory (CCR7^{-}CD45RO^{+}), Terminally Differentiated (CCR7^{-}CD45RO^{-}); **(C)** Tfh (CCR7^{+}CD45RO^{+}CXCR5^{+}), Treg (CCR7^{-}CD45RO^{+}CD25^{+}CD127^{low}) and activated CD8+ T-cells.

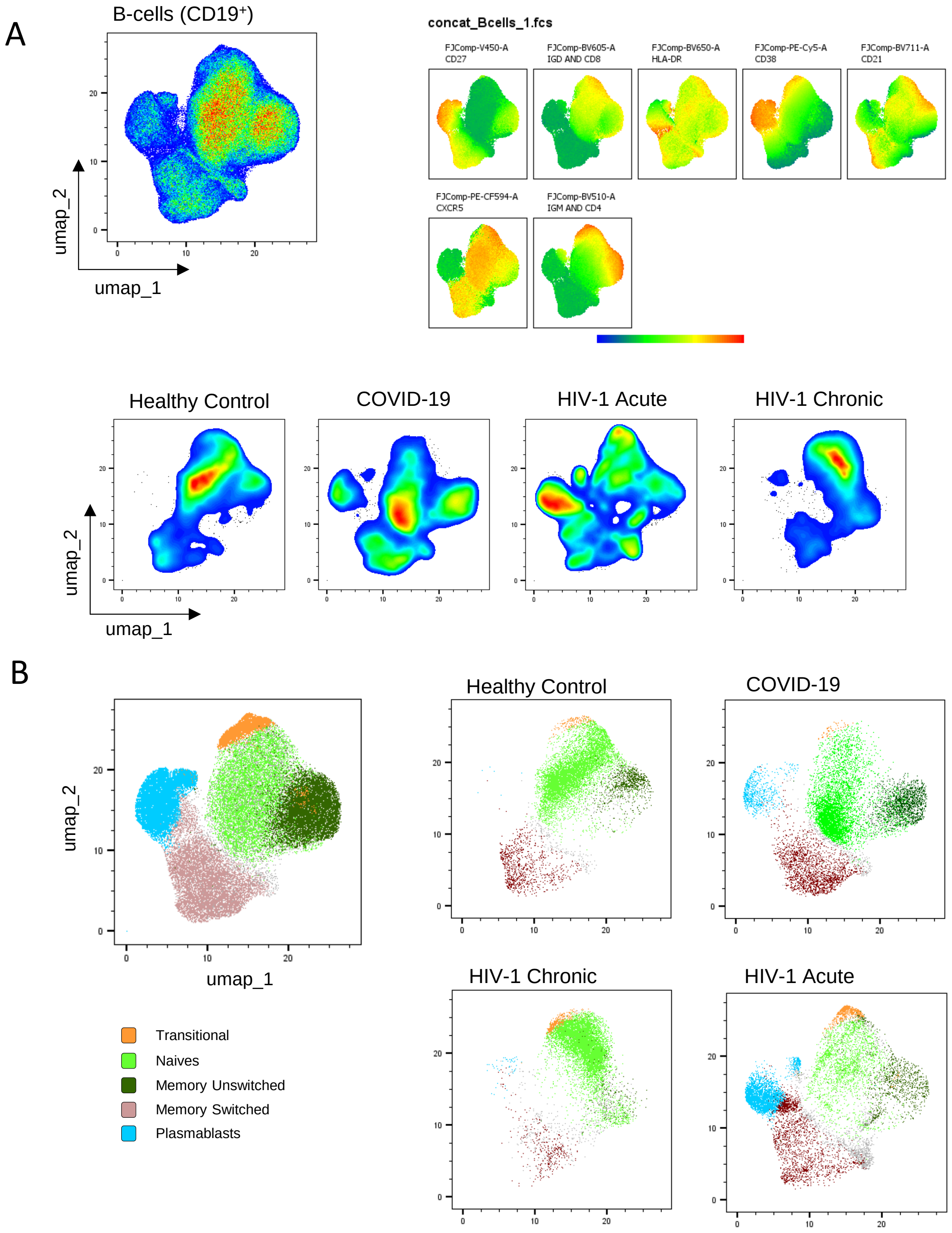

Figure S4. *Unsupervised analysis of B-cells*. **(A)** Marker expression within the UMAP of the CD19^{+} cells and relative event distribution representative individual from the healthy control and COVID-19 groups, as well as in the untreated HIV-1 acute and HIV-1 chronic patients. **(B)** Manually annotated B-cell subsets : Transitional (CD38^{high}IgM^{high}), Naïve (IgD^{+}IgM^{+}CD27^{-}), Memory Unswitched (IgD^{+}IgM^{+}CD27^{+}), Memory Switched (IgD^{-}IgM^{-}CD27^{+}), Plasmablasts (CD38^{high}CD27^{high}IgM$^{-/dim}$).

Table S1. Flow-cytometry antibodies

Antibody	Fluorochrome	Clone	Source
CD27	BV421	M-T271	BD Horizon
Vio Blue	Vio Blue	DD-1	Milteny Biotec
CD141	BV510	1A4	BD Biosciences/BD Horizon
IgM	BV510	MHM-88	Biolegend
CD4	V500	RPA-T4	BD Horizon
CD45	BV605	HI30	BD Biosciences
IgD	BV605	IA6-2	Biolegend
CD8α	BV605	RPA-T8	Biolegend
HLA-DR	BV650	L243	Biolegend
CD86	BV711	IT2.2	Biolegend
CD21	BV711	B-ly4	BD Horizon
PD-L1	BV785	29E2A3	Biolegend
CD45RO	BV785	UCHL1	Biolegend
CD3	Fitc	αT3	eBioscience/Invitrogen
CD19	Fitc	HB19	eBioscience/Invitrogen
CD66b	Fitc	G10FS	Biolegend
CCR7	FITC	150503	R&D Systems
CD14	Percp Cy5.5	61D3	eBioscience/Invitrogen
CD3	PerCP Cy5.5	OKT3	eBioscience
CD80	Pe	2D10	Biolegend
CD56	PE	C5.9	Cytognos
CD163	Pe Dazzle-594	GH1/61	Biolegend
CXCR5	PE Dazzle594	J252D4	Biolegend
CD206	Pe-Cy5	15-2	Biolegend
CD38	PE-Cy5	HIT2	Biolegend
CD123	Pe-Cy7	6H6	eBioscience
TCR-γδ	PE-Cy7	11F2	BD Biosciences
CD19	PE-Cy7	J3-119	Beckman Coulter
CD25	APC	2A3	BD Biosciences
CD204	Apc	7C9C20	Biolegend
CD16	Af700	368	Biolegend
CD1c	Apc-Cy7	L161	Biolegend
CD127	APC-ef780	eBioRDR5	Invitrogen
